# Simultaneous Fractures of Distal and Proximal Ends of Radius and Ulna: Four Fractures in One Forearm

**DOI:** 10.7759/cureus.23097

**Published:** 2022-03-12

**Authors:** Majdi Hashem, Reem A AlMohaini

**Affiliations:** 1 Department of Surgery, Faculty of Medicine, Imam Mohammad Ibn Saud Islamic University, Riyadh, SAU; 2 Department of Surgery, Faculty of Medicine, ‏Imam Mohammad Ibn Saud Islamic University, Riyadh, SAU

**Keywords:** forearm, ulna, radius, proximal, distal, fractures

## Abstract

A few cases reported simultaneous ipsilateral distal and proximal forearm fractures. This case report highlights a rare mechanism of injury that occurred due to extended forearm undergoing forced hyper-supination without the presence of ligamentous injury and the use of flexible fixation. A 49-year-old male truck driver presented to the emergency department as a motor vehicle accident (MVA) patient. A radiographic examination revealed a right forearm fracture with proximal and distal bone fractures. The distal radius was stabilized with three Kirschner wires (K-wires), and the radial neck fracture was stabilized with a single intramedullary K-wire. The olecranon and distal ulna fractures were also fixed with two intramedullary wires. On the eighth week after surgery, all K-wires were removed, and the fiberglass splint was reapplied for another two weeks. After the cast was removed, physical rehabilitation began. During the fourth month, a follow-up radiograph revealed complete healing and full wrist range of motion (ROM) with good hand grip.

## Introduction

The forearm is made up of the ulna, which is generally straight, and the radius, which is bent. The elbow joint capsule and the annular ligament bring these bones together proximally at the proximal radioulnar joint. The distal radioulnar joint (DRUJ), which is maintained by the triangular fibrocartilage complex (TFCC), is where they come into contact. The interosseous ligament, or membrane, runs between the bones and serves to keep them in their proper alignment, with a strong central band providing the main support [1]. The forearm is an important part of the human body that allows us to carry out our daily tasks. Furthermore, they are considered as a single unit. As a result, each forearm fracture should be evaluated for the radioulnar joint, which is usually injured [2,3]. The treatment of choice for displaced fractures of the radius and ulna in adults is open reduction and internal fixation. Closed reduction is designated for those who are unable to undergo surgery, and unsatisfactory results are possible up to 71% of the time. For contaminated open fractures and fractures with substantial soft-tissue deterioration, external fixators may be recommended. In adults, intramedullary nailing is rarely utilized. Dislocations associated with Galeazzi or Monteggia fractures are frequently reduced by anatomic reduction of the fracture. Fracture reduction should be reconsidered if the joint is not reducible; soft-tissue interposition may need open reduction. A radioulnar pin may be utilized to hold reduction if the DRUJ is unstable after anatomic reduction [1].

Although fractures to this bone are prevalent, Monteggia and Galeazzi fracture-dislocations are rare, unstable forearm injuries [4,5]. Monteggia is a proximal ulna fracture with radial head dislocation, whereas Galeazzi is a distal radial shaft fracture with disruption of the DRUJ [3,6]. Hyper-pronation of the extended forearm was also identified as the most common mechanism of injury [5,7]. This was not the case with our 49-year-old patient, who suffered a complicated ipsilateral four-site fracture in both the ulna and the radius, proximally and distally, without ligament involvement, as a result of forceful extension and hyper-supination during a motor vehicle accident (MVA).

## Case presentation

**Figure 1:**
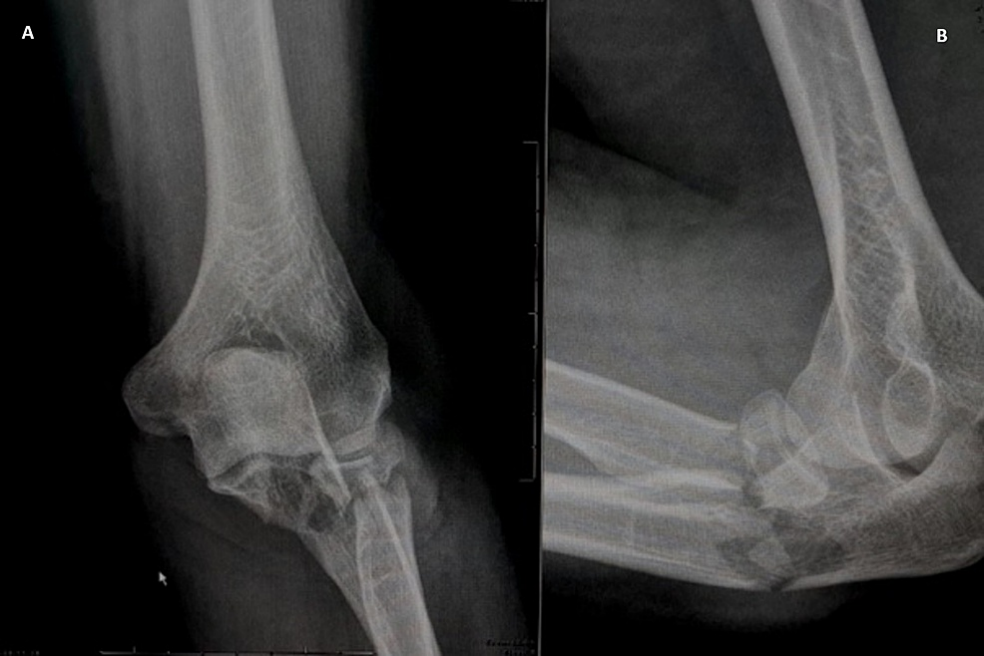
Fractures at the proximal level. Anteroposterior (A) and lateral (B) radiography illustrating proximal ulna fracture with comminution and a radial neck fracture.

**Figure 2:**
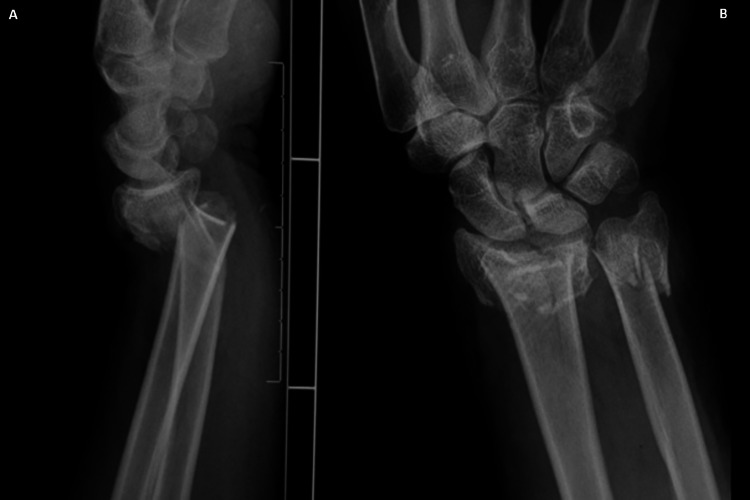
The fracture at the distal level. Anteroposterior (A) and lateral (B) radiographs showing distal radius and ulna metaphyseal comminuted fracture with dorsal angulation and displacement.

A 49-year-old male truck driver presented to the emergency department as an MVA patient. He had pain, swelling, and a severe deformity in his right forearm when he presented. The primary survey revealed a hemodynamically stable patient who was completely awake (Glasgow Coma Scale score of 15/15) and breathing spontaneously. While going on to the secondary survey, the patient provided an AMPLE (allergy, medications, previous medical history or illness, last meal, and events related to injury) history, stating that the event occurred when his right hand became entrapped within the steering wheel and twisted, followed by a fast brake and deceleration. Furthermore, the patient stated that he had no medical conditions. An isolated right forearm injury was detected during a physical examination, along with a substantial swelling of the right forearm, an apparent wrist deformity, and an intact distal neurovascular. A radiographic examination revealed a right forearm fracture with proximal and distal bone fractures (Figures 1, 2).

**Figure 3:**
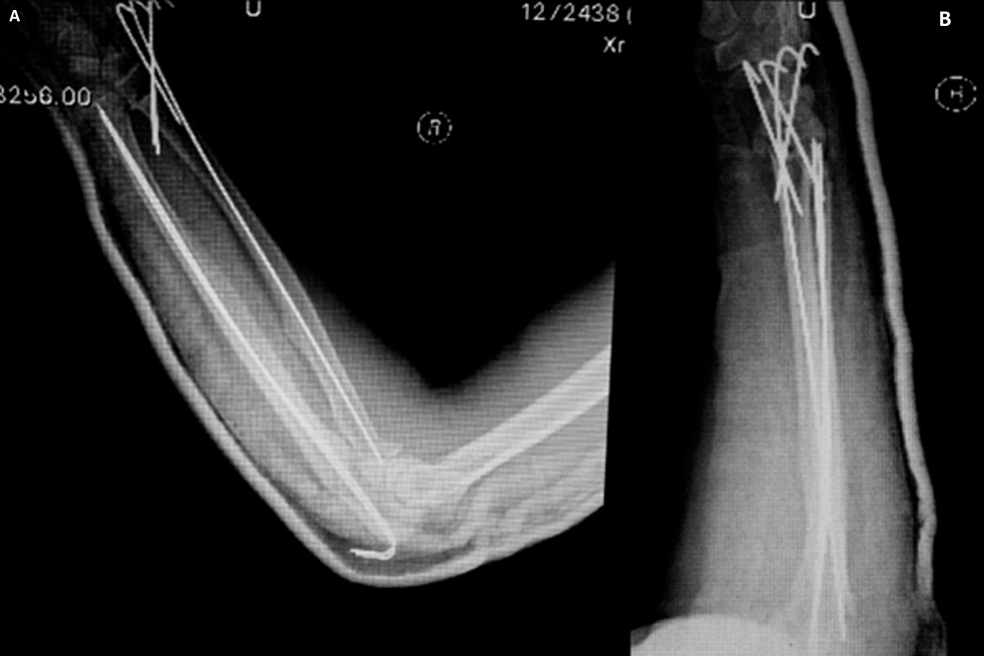
Postoperative anteroposterior (A) and lateral (B) radiographs of the forearm showed a good reduction and restored alignment. Three K-wires were used for the distal radius, and one intramedullary K-wire was used to fix the radial neck fracture. Two intramedullary wires were used to fix the olecranon and distal ulna fractures.

The patient was admitted and a splint was fitted in preparation for surgery. A surgical consent for a closed vs. open reduction with internal fixation was also signed by the patient. The patient was ushered into the operating room after everything had been prepared and consent had been obtained. Closed reduction was achieved intraoperatively under image intensifier control. The distal radius was stabilized with three Kirschner wires (K-wires), and the radial neck fracture was stabilized with a single intramedullary K-wire. In addition, the olecranon and distal ulna fractures were fixed with two intramedullary wires. As a result, as indicated in Figure 3, the decrease was achieved and alignment was restored.

**Figure 4:**
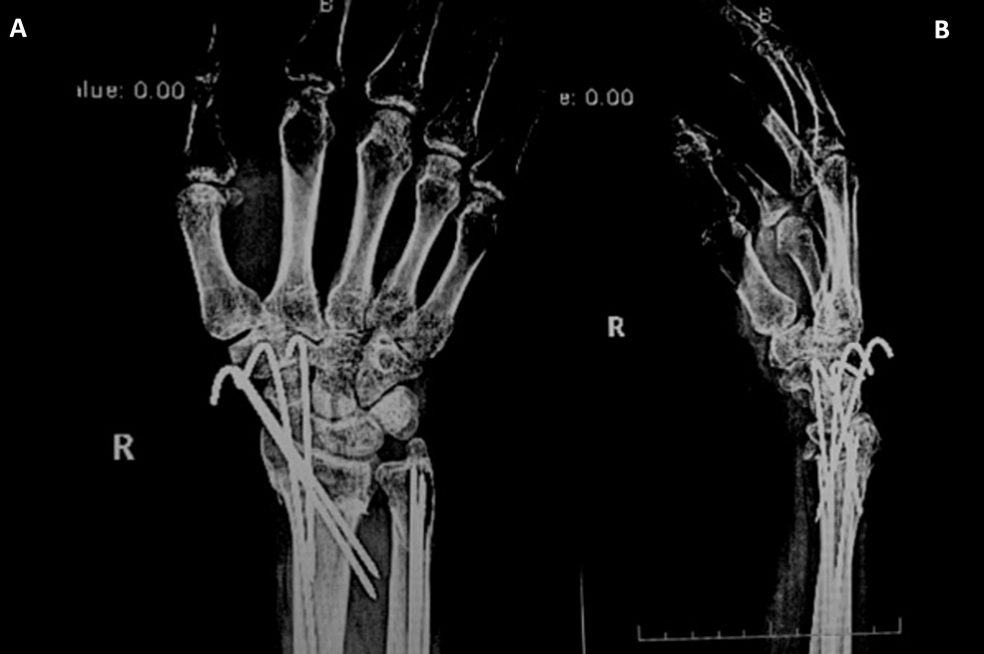
Anteroposterior (A) and lateral (B) wrist and distal forearm radiography done six weeks postoperatively. Out of splint radiographs showing excellent healing with callus formation and alignment maintained (radial height, volar inclination).

**Figure 5:**
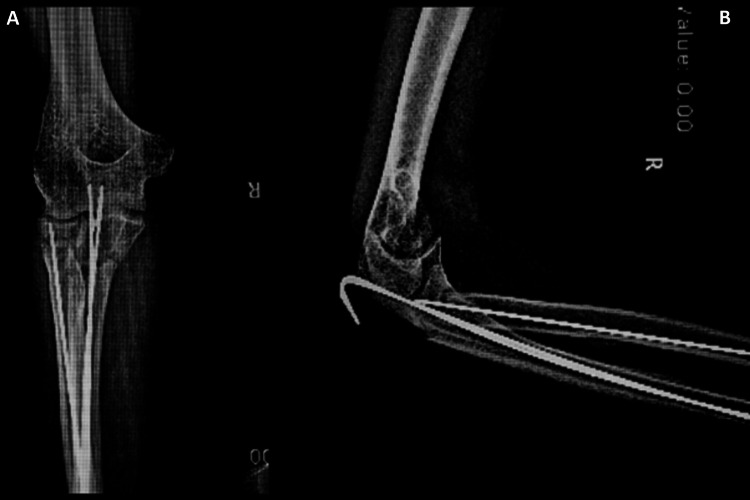
Anteroposterior (A) and lateral (B) elbow and proximal forearm radiography done six weeks postoperatively. Out of splint radiographs showing good healing process and alignment maintained.

All K-wires were bent, and the edges were placed outside the skin. A follow-up at week six revealed a healthy healing process with callus formation, as well as alignments that were kept within acceptable ranges (radial height, radial length, and volar inclination) (Figures 4, 5).

**Figure 6:**
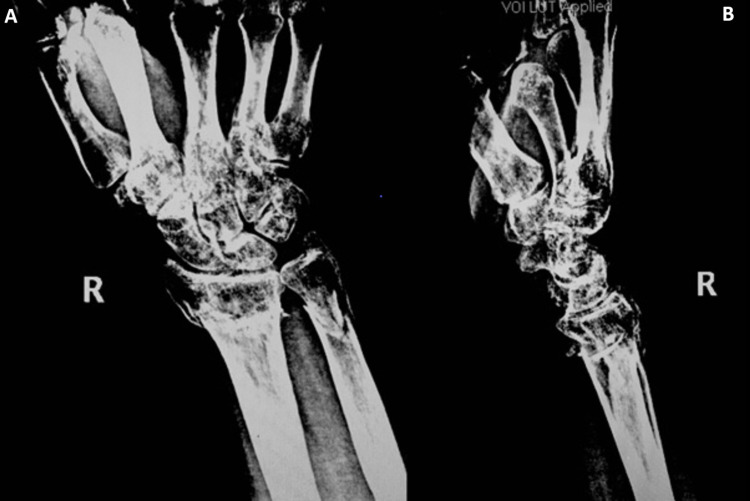
Anteroposterior (A) and lateral (B) wrist and distal forearm radiography done 12 weeks postoperatively. Radiographs showing good healing and alignment maintained (radial height, volar inclination).

**Figure 7:**
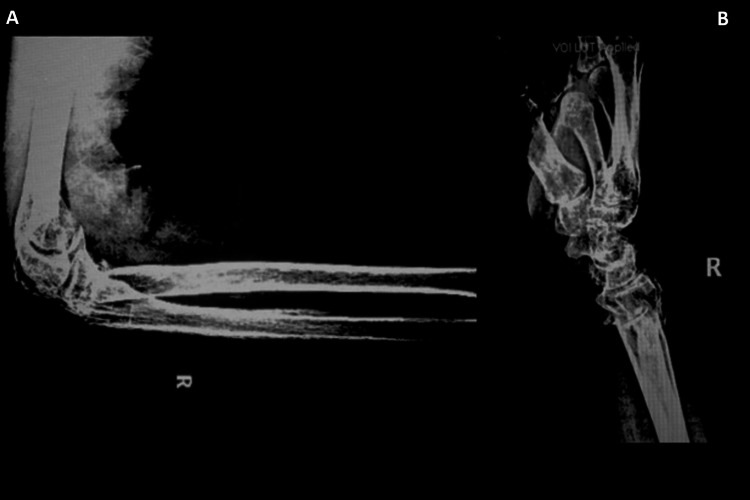
Anteroposterior (A) and lateral (B) elbow and proximal forearm radiography done 12 weeks postoperatively. Radiographs showing good healing and alignment maintained.

On the eighth week after surgery, all K-wires were removed, and the fiberglass splint was reapplied for another two weeks. After the cast was removed, physical rehabilitation started. Physical therapy was continued for an additional two months to allow a full range of motion and restore the power of the biceps and triceps. A follow-up during the fourth month revealed complete healing on radiographs and full wrist range of motion (ROM) with good hand grip; elbow ROM was 10 to 100 degrees (Figures 6, 7).

Anatomical restoration is essential to function, just as any forearm shaft fracture is regarded as comparable to an articular fracture. Internal fixation through open reduction was planned; soft tissue status has to be taken into account. Closed reduction of the distal radius fracture was done using three K-wires, followed by intramedullary elastic nail fixation of the proximal and distal ulna, and, finally, Métaizeau technique elastic nail fixation of the radial neck fracture. The reduction was feasible. No tourniquet was applied in our case. The procedure took 40 minutes.

## Discussion

The following are the main lessons to be learned from our case report: a preoperative re-evaluation of soft tissue by removing the splint prior to anesthesia is recommended, especially with closed fractures and considering the extent of trauma and the mechanism of injury; closed fractures with severe soft-tissue compromise should be delayed if necessary; and the standard method of managing such fractures is open reduction and internal fixation to ensure stability, healing, and faster rehabilitation.

The majority of the literature discusses the complications of ipsilateral radius and ulnar fractures. A similar mechanism of injury to such a case has yet to be reported in the literature. Furthermore, complex forearm Monteggia and Galeazzi lesions have been seen in rare patients. Complex forearm Monteggia and Galeazzi lesions had a 1-2% and 3-6% incidence rate, respectively [4,5]. Simultaneous ipsilateral occurrences of such a complex injury, on the other hand, are uncommon in both children and adults [4-13]. The radial head was present in this case; nonetheless, according to the Bado classification [13,14], this injury cannot be classified as a Monteggia-equivalent fracture. On the distal level, the injury was secondary to an axial loading affecting the bone at the metaphyseal level; yet, there was no associated DRUJ dislocation or subluxation on the radiograph. Because the fracture did not affect the base of the ulnar styloid, which can make the DRUJ unstable, we thought it was not disrupted. There has never been a report of a double-level ulna and radius forearm fracture without ligamentous damage or joint instability. We paid special attention to our patient's mechanism of injury, which was caused by forced hyper-supination of the extended forearm, resulting in proximal injury and distal injury due to axial loading. The forces were passed through the radial neck and metaphyseal region of the proximal ulna, locking the radial head and protecting it from dislocation. According to a review of the literature, hyper-pronation of the extended forearm was the most common mechanism of injury for complicated forearm injuries [6,7,15]. Furthermore, rather than using an open reduction as suggested in some previous literature, the patient was managed with a closed reduction, which resulted in a good outcome with a full ROM and alignment. Finally, this case indicates the existence of a complex four-site radius and ulnar fracture, which can resemble Monteggia and Galeazzi lesions in the absence of ligamental involvement. The presence of one of these injuries in a patient is not always due to hyper-pronation of the extended forearm, as previously suggested in the literature.

## Conclusions

This case indicates the existence of a complex four-site radius and ulnar fracture, which can resemble Monteggia and Galeazzi lesions in the absence of ligamental involvement. The presence of one of these injuries in a patient is not always due to hyper-pronation of the extended forearm, as previously suggested in the literature.
